# Communication about Prognosis during Patient-Initiated Second Opinion Consultations in Advanced Cancer Care: An Observational Qualitative Analysis

**DOI:** 10.3390/ijerph18115694

**Published:** 2021-05-26

**Authors:** N. C. A. van der Velden, M. B. A. van der Kleij, V. Lehmann, E. M. A. Smets, J. M. L. Stouthard, I. Henselmans, M. A. Hillen

**Affiliations:** 1 Amsterdam University Medical Centers, Department of Medical Psychology, University of Amsterdam, Meibergdreef 9, 1105 AZ Amsterdam, The Netherlands; m.b.vanderkleij@amsterdamumc.nl (M.B.A.v.d.K.); v.lehmann@amsterdamumc.nl (V.L.); e.m.smets@amsterdamumc.nl (E.M.A.S.); i.henselmans@amsterdamumc.nl (I.H.); m.a.hillen@amsterdamumc.nl (M.A.H.); 2 Amsterdam University Medical Centers, Amsterdam Public Health Research Institute, University of Amsterdam, VU University Amsterdam, Van der Boechorststraat 7, 1081 BT Amsterdam, The Netherlands; 3 Cancer Center Amsterdam, Amsterdam University Medical Centers, University of Amsterdam, VU University Amsterdam, De Boelelaan 1118, 1081 HV Amsterdam, The Netherlands; 4 Department of Medical Oncology, Netherlands Cancer Institute, Plesmanlaan 121, 1066 CX Amsterdam, The Netherlands; j.stouthard@nki.nl

**Keywords:** advanced cancer, palliative care, medical decision making, second opinions, referral and consultation, quality of care, prognosis, truth disclosure, physician-patient communication, physician-patient relations

## Abstract

Prognostic communication is essential for patients with advanced cancer to enable informed medical decision-making and end-of-life planning. Discussing prognosis is challenging, and might be especially complex for oncologists conducting a second opinion (SO). Survival data are often lacking, and consulting oncologists need to consider previously conveyed information and patients’ relationship with the referring oncologist. We qualitatively investigated how advanced cancer patients and consulting oncologists discuss prognosis during audio-recorded SO consultations (*N* = 60), including prognostic information received from the referring oncologist. Our results show that patients regularly expressed implicit cues to discuss prognosis or posed explicit questions tentatively. Consulting oncologists were mostly unresponsive to patients’ cues and cautious to prognosticate. They also seemed cautious when patients brought up the referring oncologist. Consulting oncologists checked which prognostic information patients had received from the referring oncologist, before estimating prognosis. They agreed with the first opinion or rectified discrepancies carefully. Altogether, this study exposes missed opportunities for open prognostic discussions in SOs. Consulting oncologists could explicitly explore patients’ information preferences and perceptions of prognosis. If desired, they can provide tailored, independent information to optimise patients’ prognostic awareness and informed medical decision-making. They may additionally support patients in dealing with prognosis and the uncertainties associated with it.

## 1. Introduction

Cancer patients are confronted with a life-threatening disease, leading to considerable distress [1]. For many patients, the diagnosis involves complex decision-making about treatment and substantial uncertainty regarding their prognosis, i.e., the (in)curability of the disease and life expectancy [2,3,4,5,6]. Information about prognosis generally includes a high degree of uncertainty, as the precision of prognostic estimates and their predictive values for individual patients are limited [7,8]. In an era of rapidly developing anti-cancer therapies with high survival benefits for only a subset of patients, prognostication is even further complicated [9,10].

Nevertheless, prognostic information is assumed to be essential for cancer patients’ medical decision-making, as it informs the trade-off between risk and benefit, and the balance between length and quality of life [10,11,12]. Prognostic information might be particularly important for patients with advanced disease because it allows them to make tailored choices about palliative treatment and end-of-life issues [13,14,15,16]. Previous research among patients with advanced cancer shows that increased awareness of prognosis is associated with less aggressive therapy and higher quality of life. In contrast, prognostic unawareness might lead to decisions conflicting with patients’ actual values and unsatisfactory disease management [15,17,18]. 

The importance of prognostic communication is generally recognised and the majority of advanced cancer patients prefer prognostic disclosure [19,20,21]. Yet, studies indicate that oncologists rarely disclose life expectancy [8,9]. Contributing to oncologists’ reluctance may be fears of being inaccurate, sometimes driven by experiences of seeing predictions turn out wrong [22,23]. Indeed, the accuracy of oncologists’ predictions varies widely, with a general tendency towards overestimation of survival [24,25,26]. Fears of upsetting patients, taking away hope, or damaging the physician-patient relationship may additionally explain oncologists’ hesitance [8,10,27,28,29]. Accordingly, in previous research involving consultations about palliative chemotherapy, patients rather than oncologists were found to take the initiative to discuss prognosis [8,30]. If disclosed, oncologists often emphasise the uncertainty of prognostic estimates and use ambiguous rather than precise terms (e.g., “months to years” versus average or median survival) [8,30]. 

The obstacles of communicating prognosis to advanced cancer patients could be even higher for oncologists conducting second opinions (SO). SOs are medical re-assessments by an independent (i.e., consulting) physician from the same specialty as the treating (i.e., referring) physician [2,6,31,32,33]. In SO consultations, oncologists’ prognostication might be compromised by yet unknown data on success and survival for the opted experimental treatment modalities. Furthermore, the involvement of a second physician induces complexity in the communication. For patients, it might be difficult to interact with two oncologists and process potentially distinct information [34]. In turn, consulting oncologists need to take into account the patient’s relationship as well as their own professional connection with the referring oncologist [35,36]. This calls for objectivity, diplomacy and a careful choice of words. Previous literature shows that most consulting oncologists refrain from disclosing minor medical discrepancies to avoid disagreeing with the first opinion and confronting patients with inconsistent information [35,37,38]. Contradictions could cause uncertainty, confusion, and distress among patients or diminish their trust in the referring oncologist [2,3,31,35]. The risk of conveying inconsistent information might be increased when discussing prognosis, as its uncertain nature and oncologists’ ambiguous language leave room for interpretation. Yet, since we know a subset of patients seek SOs for additional prognostic information, patients may be under-informed or disappointed if the prognosis is not discussed at all [2,39,40,41,42]. 

To date, few studies have investigated the content of SOs and their communicative aspects; none have investigated how prognosis is discussed [2]. Given the relevance of prognosis for advanced cancer patients, we need to gain insight into how prognostic communication takes place in the challenging setting of patient-initiated SOs. Therefore, this study aimed to explore (1) how advanced cancer patients phrase their questions and ideas about prognosis; (2) how consulting oncologists discuss prognosis with advanced cancer patients; and (3) how advanced cancer patients and consulting oncologists discuss prognostic information previously provided by the referring oncologist. 

## 2. Materials and Methods

### 2.1. Study Design

The presented qualitative analysis was part of a larger prospective mixed-methods study about patient-initiated SOs in medical oncology (SO-COM), which examined communication during SOs and their psychological impact on patients [31]. The SO-COM study included audio recordings of SO consultations and self-report surveys from patients, referring oncologists and consulting oncologists (for more detailed information, see Lehmann et al. [31]). For the current analysis, we used the audio recordings of the SO-COM study to assess prognostic discussions by advanced cancer patients and consulting oncologists.

### 2.2. Recruitment and Sample

Medical oncologists affiliated with two Dutch tertiary referral centres were informed about the SO-COM study and invited to participate. Consecutive cancer patients scheduled for a SO with consenting oncologists were informed by hospital staff and consequently contacted by the study team. Both consulting oncologists and patients provided written informed consent for participation. 

Patients scheduled for a SO were eligible for the SO-COM study if they had any type of solid tumour, were older than 18 years and had sufficient Dutch language proficiency. The SO-COM study originally comprised audio recordings of 69 patients. For the present analysis, we selected recordings of patients with advanced cancer stages, resulting in *N* = 60 consultations.

All procedures were in accordance with the Helsinki Declaration and were approved by the institutional medical ethics review board of the study contractor (Amsterdam University Medical Centres, NL63087.018.17; study number 2017_271) and the local review boards of both study sites. 

### 2.3. Data Collection

The Standards for Reporting Qualitative Research were followed [43]. All audio recordings were fully listened to and relevant segments were anonymously transcribed verbatim. Relevant segments of the consultation involved (1) direct discussion of prognosis (i.e., (in)curability, life expectancy or the word “prognosis”) or (2) communication about prognosis-related topics (e.g., life extension, survival gain, delay of tumour growth, treatment duration and effectiveness). The latter category was included because it may contain implicit information about prognosis, thereby indirectly affecting patients’ prognostic awareness or triggering discussions of (in)curability or life expectancy. Relevant segments of significant others accompanying advanced cancer patients were included as well since these influenced prognostic communication during the consultation. All transcripts were imported into the coding software MAXQDA2020 [44].

### 2.4. Analysis 

A qualitative analysis was performed using an inductive coding approach to allow for new themes to arise [45]. Data analysis was conducted by MK (last year medical student, trained in qualitative methods), NV (psychologist and researcher with expertise regarding prognostic communication, trained in qualitative methods) and MH (psychologist and researcher with expertise regarding second opinions and qualitative methods). NV and MK independently coded fifteen transcripts to enhance data triangulation, using open coding [46]. After every five transcripts, results were jointly discussed by NV, MK and MH to reach a consensus on an initial coding scheme. Throughout this process, the coding scheme was further refined and hierarchical layers emerged. Previously analysed transcripts were reviewed to make any necessary coding adjustments based on the updated coding scheme. When only a few new codes were added during the third consensus discussion, the coding scheme was finalised. MK coded all remaining 45 transcripts. In addition to segments that MK perceived as ambiguous, one out of every five transcripts was double-coded by NV and discussed to reach an agreement. After completion of the open coding process, prominent topics were derived from the final coding scheme. All three authors reviewed these to generate higher-order descriptive themes of prognostic communication in SOs for advanced cancer patients. Extensive discussions further explored potential links between recurring themes. Illustrating quotes were identified from the transcripts throughout the data analysis to substantiate findings.

## 3. Results

Twenty-one different oncologists (57% female, *n* = 12/21) consulted with 60 advanced cancer patients, who were mostly female (62%, *n* = 37/60) and aged between 28 and 85 years (*M* = 58). Most common cancer types were gastrointestinal cancer (32%, *n* = 19/60), breast cancer (25%, *n* = 15/60), urogenital cancer (17%, *n* = 10/60) and melanoma (12%, *n* = 7/60). All but one patient came with at least one significant other (98%, *n* = 59/60). The mean duration of the SO consultations was 39.57 min (*SD* = 12.60) and ranged from 19.11 to 72.57 min. 

Fifty-six of the analysed consultations included relevant segments (*n* = 56/60). In more than 80% of these consultations, the prognosis was discussed directly (*n* = 46/56). Of these 46, 15 involved talk about the (in)curability of the disease only, another 15 exclusively discussed life expectancy and 16 consultations included both subjects. Communication about prognosis-related topics (e.g., life extension, survival gain, delay of tumour growth, treatment duration and effectiveness) took place in nearly all consultations (*n* = 54/56). Results of the qualitative analysis are presented below, structured according to our three research questions. 

### 3.1. How do Advanced Cancer Patients Phrase Their Questions and Ideas about Prognosis during SO Consultations?

#### 3.1.1. Initiative to Discuss Prognosis

Discussions of prognosis were frequently initiated by patients or their significant others. They were often explicit about their wish for prognostic information, but still posed questions cautiously. The timing for starting these conversations varied across consultations. Several patients brought up prognosis immediately at consultation onset, suggesting that a need for more specific estimates had motivated them to request the SO (Q1). Yet, patients who had primarily sought the SO for other reasons, such as assistance in treatment decision-making, also uttered a wish for prognostic information (Q2).

Q1 Consulting oncologist: ‘I received a very nice [referral] letter, but if you had to put it in your own words: what can I do for you?’
  Patient: ‘Yes, what you can do for me… What do you think about the whole thing of, like, um, in terms of life exp… How long can I still, like, how long do I have left to live? ’
  – Consultation #1/female patient/57 years old /breast cancer
Q2 Patient: ‘What are my most important questions? Whether the suggested therapy, the chemotherapy, is the right one. For my family, it’s most important to find out whether I would qualify for immunotherapy. And do I have any other matters to discuss…? Yes, we would like to have some kind of indication in terms of time.’
  – Consultation #2/male patient/53 years old/stomach cancer

Other patients and their significant others initiated a conversation about life expectancy over the course of the consultation. Some additionally mentioned observing consulting oncologists’ caution in discussing prognosis (Q3), which might indicate awareness of how difficult such assessments are for oncologists. A few patients started a prognostic discussion towards the end of the consultation (Q4).

Q3 Significant other: ‘Speaking of time, because you’re very reserved about that, otherwise you would have said something. Can you, can you give us some kind of indication, or...?’
  – Consultation #3/male patient/75 years old/rectal cancer
Q4 Consulting oncologist: ‘Do you have any more questions or are there things we haven’t discussed yet?’
  Patient: ‘Um, yes. I still want to ask you, how much time do I have left to live?’
  – Consultation #4/female patient/66 years old/ovary cancer

#### 3.1.2. Implicit Cues and Hope for Positive Outcomes 

Rather than posing explicit questions about prognostic estimates, patients and their significant others often seemed to pursue a conversation about prognosis implicitly, by mentioning hope for optimistic scenarios or participation in future events. They did not ask for (dis)confirmation, nor did they subsequently request specific prognostic information. Consulting oncologists mostly did not respond to such implicit cues about prognosis (Q5).

Q5 Significant other: ‘Yes, she is sick, but she feels relatively well. And we hope we can go on like that for 40 to 50 years, but we don’t know that...’
  Consulting oncologist: ‘Yes, and what do you do, apart from working?’
  – Consultation #5/female patient/53 years old/breast cancer

Some patients seemed to draw hope for better outcomes from other patients’ exceptional treatment response and survival. Consulting oncologists counterbalanced such hope by sketching more realistic scenarios, based on group statistics instead of individual cases (Q6).

Q6 Patient: ‘And then you read these sensational stories [about experimental treatment abroad] from patients who live for another 9 years. Those stories make you want that too.’
  Consulting oncologist: ‘Yes, positive stories may be told as well, but those are mostly about one individual. If they would be about a whole lot of people […] we should be asking ourselves whether we [as oncologists] are doing it right in The Netherlands. […] I can imagine it gives you hope on the one hand, but on the other hand, you could think, “What does it really mean, what am I supposed to do with that?”’
  – Consultation #6/female patient/61 years old/brain cancer

### 3.2. How do Consulting Oncologists Discuss Prognosis with Advanced Cancer Patients during SO Consultations?

#### 3.2.1. Cautiousness about Discussing Prognosis 

In a few cases, consulting oncologists took the initiative to discuss prognosis with advanced cancer patients. When they did, it pertained to the incurability of the disease. Regarding life expectancy, oncologists were generally reluctant to provide estimates when patients asked for it. They additionally stressed the indeterminacy of prognosis (Q7 and Q8).

Q7 Consulting oncologist: ‘Well, and as for the future, the difficult thing is that crystal ball [to foresee the future]. They don’t have one in [the referring hospital], but I don’t have it here either.’
  – Consultation #7/male patient/55 years old/kidney cancer
Q8 Consulting oncologist: ‘Yes, that is always so difficult, because of course we can’t tell you what your life expectancy is. That’s so difficult. As doctors, we’re actually not that good at estimating life expectancy. Certainly not when the patient sitting in front of us is in a good condition. When patients are hospitalised and they’re very ill, we could say, “This is not going to last longer than a few days”. We can do that. But everything else, we can’t. So I never do [provide estimates]. I also just don’t know.’
  – Consultation #8/female patient/41 years old/melanoma

Consulting oncologists commonly explained the indeterminacy of prognosis by pointing out the influence of other uncertain factors, such as treatment effectiveness. Others expressed concerns about being held responsible for their prognostic statements (Q9).

Q9 Consulting oncologist: ‘The difficulty of communicating prognosis is that it will start living a life of its own. The doctor specifies a certain amount of time and then it’s always like, “But the doctor said this and in the end, it turned out to be that”.’
  – Consultation #9/female patient/60 years old/breast cancer

In a few consultations, consulting oncologists more strongly expressed their apprehension about prognostication in late-stage cancer, which they suggested would reflect medical incompetence (Q10). These comments may relate to the lack of survival statistics for advanced cancer generally, and for patients receiving multi-line therapy or surpassing prognostic estimations specifically.

Q10 Consulting oncologist: ‘Well, it’s like that indeed: we can’t say anything. You can see that looking at your entire disease history. The fact that someone is still responding to a treatment so well after 2 years, 2.5 years even, is unique already. The way you are right now, that’s unique. Um, so in other words, you should disregard everyone who would predict something right now and view them as incompetent.’
  – Consultation #10/female patient/36 years old/breast cancer

#### 3.2.2. Generic Terminology and Disclaimers for Precise Estimates

When consulting oncologists did provide information about life expectancy, they mainly used imprecise terms that were open to interpretation (Q11 and Q12). For example, they used words like “short” or “fast” to express some sense of time, as well as wide non-numerical time frames (e.g., “months to years”).

Q11 Consulting oncologist: ‘But cancer still is an unpredictable disease. If you do the things that we’re doing right now, yes, then life expectancy could be long. And if the chemotherapy does not work, then it might be shorter.’
  – Consultation #2/male patient/53 years old/stomach cancer
Q12 Consulting oncologist: ‘When I look at you right now: you’ve got a lot of options left, the tumour doesn’t seem to grow very fast, it responded to the chemotherapy. So I think that, in that regard, you’ve still got a lot of time. Although it will always be too short.’
  – Consultation #11/female patient/62 years old/colon cancer

A few consulting oncologists indicated what patients’ life expectancy would definitely *not* be, rather than estimating their actual prognosis (Q13). This seems to accentuate the inherent difficulty of providing exact, definite estimates.

Q13 Consulting oncologist: ‘None of us will be talking about years [time until patient’s death]. No. I just don’t believe that. And more than that I can’t say right now. That’s not because I don’t want to, but because I don’t know.’
  – Consultation #1/female patient/57 years old/breast cancer

In a small minority of the consultations, consulting oncologists communicated precise and/or numerical prognostic information upon patients’ requests (e.g., average or median survival), always accompanied by disclaimers. They underscored the generic nature of estimates and expressed reservations about the applicability of group statistics to individual patients (Q14).

Q14 Consulting oncologist: ‘Well, what I can tell you–but I will just speak in very generic terms–so once again, I don’t know if that applies to your wife… If you would do nothing at all, then the life expectancy is, on average, for colorectal cancer in general, 6 to 7 months.’
  – Consultation #11/female patient/62 years old/colon cancer

#### 3.2.3. Emphasis on Positive Scenarios

Some consulting oncologists added information about positive and/or negative exceptions to the average or median life expectancy. When doing so, they often specifically emphasised the positive outliers in an effort to foster hope (Q15). None of the consulting oncologists in our sample exclusively mentioned the worst-case scenario.

Q15 Consulting oncologist: ‘What’s important to know is that there’s a certain average, or we actually call that the median. […] On average, it’s 2 to 3 years. But there are women who are still alive after 15 years. And there are women who deteriorate very fast. Then it will be shorter. Most patients remember those 2 to 3 years, but hold on to the exceptions. […] You should hold on, I think, to those people who live for a long time with a good quality of life.’
  – Consultation #9/female patient/60 years old/breast cancer

#### 3.2.4. Discussion of Prognosis-Related Topics

Apart from discussions about (in)curability and life expectancy, nearly all analysed consultations involved communication about topics strongly related to prognosis. This concerned issues such as life extension, survival gain, delay of tumour growth, treatment duration and its effectiveness. Consulting oncologists appeared less reluctant to provide numerical information about these subjects as compared to life expectancy (Q16 and Q17).

Q16 Consulting oncologist: ‘So it is possible… There are patients who still benefit from it [chemotherapy] after 6, 7, 8, 9, even 10 years.’
  – Consultation #12/female patient/58 years old/melanoma
Q17 Consulting oncologist: ‘My first advice is to use the therapies that have proven useful, chemotherapy and such, as you will live 4 times longer with chemotherapy than without it.’
  – Consultation #13/female patient/48 years old/colon cancer

When discussing prognosis-related topics, consulting oncologists did not explain the implications for patients’ (in)curability or life expectancy. Yet, some patients appeared to infer information about their prognosis from these related topics (Q18). A few times, this subsequently triggered a conversation about prognosis.

Q18 Consulting oncologist: ‘Your life will be divided into periods of 2 months. You will get 3 chemotherapies and a week of pills. That will be repeated 3 times before you get the next CT scan. Then we’ll decide whether we’ll give you another 3 cycles. After 6 cycles we will take a break.’
  Patient: ‘Um... So I’m holding on to the 6x2 months of treatment, then we’ll be a year further already.’
  – Consultation #2/male patient/53 years old/stomach cancer

### 3.3. How do Advanced Cancer Patients and Consulting Oncologists Discuss Prognostic Information Provided by the Referring Oncologist?

#### 3.3.1. Patients’ Communication about Prognostic Information Provided by the Referring Oncologist 

About 40% of the consultations involved conversations about prognostic information previously provided by the referring oncologist (*n* = 24/56). These discussions were predominantly initiated by patients and their significant others, who sometimes simply repeated what the referring oncologist had said (Q19 and Q20). Consulting oncologists in our sample did not explicitly explore these implicit cues to talk about previous prognostic information.

Q19 Significant other: ‘We’ve known for 3 years now what’s about to happen, and from the first day on they [referring doctors] have said they don’t know when [the patient will die]. They still say that. It could take months, it could take years.’
  – Consultation #14/female patient/54 years old/colon cancer
Q20 Patient: ‘I also asked the surgeon how long I’ve got left to live. She said, “1 Year, 10 years, 20 years, that depends on how the medicine works”. Therefore, I made a call yesterday to ask for an MRI. And that’s why the scan has been ordered.’
  Consulting oncologist: ‘And why do you want that MRI?’
  – Consultation #15/female patient/61 years old/breast cancer

Other patients only mentioned that the prognosis given by the consulting oncologist matched the information they had previously received from the referring oncologist (Q21). In multiple instances, it became clear that the difficulty of prognostication had been conveyed before.

Q21 Significant other: ‘And what if she would not get the treatment? What would her prospect be?’ […]
  Consulting oncologist: ‘Um, that varies. If we would do nothing, […] then your [patient’s] condition will deteriorate. And then of course you’ll die of it [cancer] eventually. And how long “eventually” will take, nobody knows in advance.’
  Patient: ‘And that’s what the referring oncologist said as well.’
  – Consultation #1/female patient/57 years old/breast cancer

Occasionally, patients brought up the referring oncologist to express their dissatisfaction with previous prognostic communication (Q22). This most often concerned how prognosis had been disclosed, rather than the content of the information.

Q22 Patient: ‘At the start they [referring doctors] said, “You’ve got up to 5 years”. They said that at the start and one week later they said to us, “Well, maybe, 80 percent will die within one year in your case”. So I found that a little bit, yes, that was quite heavy.’
  Consulting oncologist: ‘Another setback.’
  Patient: ‘Yes, that’s a setback. I also said to that doctor, “Don’t do that anymore”. Up to 5 years, then we’re already thinking it’s going to be years, but it won’t be years according to those information booklets.’
  – Consultation #16/female patient/69 years old/oesophagus cancer

In a few consultations, patients and their significant others introduced the referring oncologist into the conversation to explicitly ask the consulting oncologist for additional prognostic information, sometimes persistently. Again, they seemed to indicate an understanding of the complexity of prognostication for oncologists (Q23).

Q23 Significant other: ‘The prognosis, well, how long he’s got left to live… Those averages were very low. We asked our oncologist about that too and she said, “Without chemotherapy it’s 3 to 5 months on average and when you do get chemotherapy, you’ll win an extra 6 to 7 months”. Um, but on average this [advanced stomach cancer] happens to 70-year-old people. So we also asked her if she could provide the outliers. Because those numbers were averages, you can’t say anything about that. Do you have any experience with that? Because he’s so young and it feels so unreal that, well, in half a year it will all be over.’
  Consulting oncologist: ‘I generally try not to mention any numbers, because that’s so difficult and it’s not predictable per individual how it will go […].’
  Patient: ‘We get that exact same reaction from our own oncologist, but it’s not like we’re expecting a doctor to say how long I’ve got left to live, you can’t answer that. […] We’ve been given an average, but this average has got a minimum and a maximum. We just want to know that minimum and maximum.’
  – Consultation #17/male patient/44 years old/stomach cancer

#### 3.3.2. Consulting Oncologists’ Communication about Prognostic Information Provided by the Referring Oncologist 

In a few cases, consulting oncologists initiated communication about the referring oncologist. Some did so to check which prognostic information had already been communicated, before disclosing the prognosis themselves. Generally, these attempts to obtain information about a previous prognosis seemed careful. In reaction to patients’ reports, consulting oncologists often supported prognostic information from the referring oncologist (Q24). Notably, one patient refused to disclose what the referring oncologist had said and specifically requested an independent prognostic assessment from the consulting oncologist (Q25).

Q24 Consulting oncologist: ‘But was anything said about whether this treatment is focused on, um, curation or on, um...’
  Patient: ‘No, he already said that it’s not curable.’
  Consulting oncologist: ‘That has already been made clear. Okay. And that’s correct, of course.’
  – Consultation #18/male patient/62 years old/prostate cancer
Q25 Significant other: ‘And what about the prognosis? Because…’
  Consulting oncologist: ‘What the prognosis is?’
  Patient: ‘Yes.’
  Consulting oncologist: ‘Yes… Has anything been said to you about the prognosis?’
  Patient: ‘Yes, but I actually want to hear it from you.’
  – Consultation #9/female patient/60 years old/breast cancer

In none of the analysed consultations did the consulting oncologist firmly contradict the referring oncologist. Consulting oncologists who expressed doubts about (parts of) the information that was previously conveyed, only exposed minor discrepancies and did so carefully (Q26). When subtly rectifying previous estimates, consulting oncologists often appeared to explain the discrepancy between both estimates (Q27).

Q26 Patient: ‘He [referring oncologist] said, “20 Years, 10 years” when I asked about it. But he also said “1 Year”. He said, “I don’t know” […].’
  Consulting oncologist: ‘Look, that’s the difficulty of it. You just don’t know. You don’t know how aggressive the cancer behaves, certainly not at this point. […] Time will have to tell how it [cancer] behaves, especially during your treatment. If you would ask right now, “Do I only have 1 year left?”, then I don’t expect that. But I also don’t know what the scan would indicate right now.’
  – Consultation #5/female patient/53 years old/breast cancer
Q27 Patient: ‘I’m just trying to compare it to what the referring oncologist said to us, “For life expectancy, you could expect half a year if you would do nothing” […].
  Consulting oncologist: ‘[…] Those averages of 6 months if you would do nothing; those are averages. I do not believe you would only have 6 months left in case you would not get any treatment. […] So that’s how he came up with that average of 6 months, that’s not strange, because it’s in all the studies we know.’
  – Consultation #3/male patient/75 years old/rectal cancer

In contrast with the aforementioned results, a few consulting oncologists disclosed life expectancy while assuming that the referring oncologist had already discussed prognosis (Q28). They did not always check if patients had been made aware of their prognosis before.

Q28 Consulting oncologist: ‘No, but I think that [the referring oncologist] has also said, “You could still have years”. It just depends on how long you’ll be stable with treatment and how fast the disease will progress.’
  – Consultation #5/female patient/53 years old/breast cancer

## 4. Discussion

### 4.1. Main Findings

This study explored how advanced cancer patients and consulting oncologists discuss prognosis during SO consultations. Our analysis revealed that patients and their significant others usually initiated prognostic communication and introduced the referring oncologist into the conversation, using explicit as well as implicit communication. Consulting oncologists were overall cautious in these discussions, as they were mostly unresponsive to patients’ cues, stressed the indeterminacy of prognosis and provided ambiguous rather than explicit estimates. When asking about previously conveyed prognostic information, consulting oncologists often supported the first opinion and rectified discrepancies carefully.

In line with previous work, the current study demonstrated advanced cancer patients’ need for prognostic information. Yet, we also exposed their struggle to start prognostic conversations straightforwardly. Patients expressed implicit cues, for example by presenting their expectations for the future and hope for positive outcomes, and posed even their explicit questions cautiously. Previous research suggests that patients commonly utter their informational and emotional needs in implicit rather than direct ways. Such indirect communication has been observed in conversations about other sensitive topics too, like alternative treatment options and death [47,48,49]. Patients’ latent communication might indicate an ambivalence towards receiving prognostic information. Especially as death is impending, it appears that patients want to be informed about their prognosis, but at the same time do not want to know [48,50,51]. They seem to recognise the practical reasons for prognostic disclosure, but fear the emotional impact of knowing [51]. Alternatively, our findings could reflect patients’ politeness, wishing not to complicate the conversation by introducing a sensitive subject [52]. Patients’ use of implicit cues might be problematic, as the literature indicates that physicians experience difficulty in detecting indirect expressions [52,53,54]. The latter was reiterated in our study. Possibly, consulting oncologists are unaware of patients’ hidden cues. Alternatively, they might not perceive an urgency to respond to implicit requests, or feel inadequate to meet patients’ prognostic information needs [48,52,55]. 

When consulting oncologists did respond to patients’ requests for prognostic information, they were particularly cautious about estimating life expectancy. Reluctance to prognostic communication has been observed in regular consultations before [8,30], indicating that barriers to discussing prognosis remain substantial across different settings. Insufficient time is often reported as a complicating factor [29,56,57], yet might be less relevant in the relatively long SO consultations in our sample (*M* = 39.57 min). Undoubtedly, prognostication is especially complex when conducting SOs in advanced cancer care. While the determination of incurability might be fairly clear-cut, overall survival varies widely and oncologists regularly overestimate individual life expectancy [26,58,59,60]. Therefore, they might not always be able to fulfil patients’ desire for certainty. In addition, personal barriers may play an important role in oncologists’ cautiousness, as previous studies showed their discomfort regarding sensitive topics. For prognosis specifically, oncologists worry about being blamed by patients, inflicting distress or failing to answer questions [48,52]. The uncertainty of estimates may further diminish their confidence in discussing this topic [59]. Hence, our results may reflect a struggle between wanting to meet patients’ needs, while avoiding definite prognostication and its feared consequences [61]. It is understandable that oncologists use strategies that might mitigate this discomfort, like ambiguous language [62]. However, this should not prevent opportunities for constructive conversations about prognosis. Interestingly, we observed that some patients expressed awareness of the difficulty of prognostication, suggesting that they might understand the limits to oncologists’ predictions. This may encourage oncologists to engage in prognostic discussions without fear that patients expect precise estimates. Additionally, several studies show no or even positive associations between prognostic disclosure and patients’ emotional well-being and the oncologist-patient relationship, which may reassure oncologists as well [18,29,63,64,65,66,67]. 

We observed that consulting oncologists were also cautious when patients mentioned prognostic information previously provided by the referring oncologist. They carefully asked patients about previous estimates before disclosing prognosis and often supported or justified prognostic statements provided by the referring oncologist. Possibly, consulting oncologists agreed with the first medical opinion. Another explanation could be that they perceived discrepancies as negligible, taking into account that minor differences may needlessly confuse or harm patients [37,38]. Alternatively, consulting oncologists might downplay inconsistent information to avoid openly disagreeing with the first opinion, thereby guarding patients’ relationship with the referring oncologist. This potentially reflects consulting oncologists’ awareness of the sensitivities of the SO setting [35,37]. Importantly, inconsistencies in prognostic estimates between the first and the second opinion may not necessarily originate from a medical disagreement between both oncologists. It is well known that patients’ recall of information may be impaired, especially in bad news consultations [68]. Moreover, patients may perceive and react to medical information differently at different stages in their disease trajectory, as each consultation changes patients’ emotions and attitudes towards their illness. Patients seeking SOs could be more “ready” to hear prognosis and therefore experience prognostic information as novel or different [37,38]. 

In contrast with the aforementioned results, we noticed less caution among consulting oncologists on a few occasions. First, some consulting oncologists disclosed life expectancy while remarking that patients had probably already heard their prognosis from the referring oncologist. Yet, they did not explicitly explore patients’ prognostic awareness beforehand. Second, consulting oncologists were less reluctant to provide numerical estimates about subjects other than life expectancy. Perhaps they were unaware of the implicit message in prognosis-related topics. Alternatively, consulting oncologists felt more comfortable in talking about, for example, treatment effectiveness, because they had more evidence available or perceived such information as less confrontational [11,48,62,69]. Nevertheless, both behaviours could confront patients with novel, possibly unwanted, yet impactful information. This may startle or upset them, and consulting oncologists should address such patient distress [61,70,71]. Furthermore, the discussion of prognosis-related topics specifically might confuse patients’ understanding of the prognosis, if not substantially clarified. Patients could silently infer prognostic information from these topics, preventing oncologists from rectifying incorrect ideas. Correspondingly, earlier research suggested that oncologists’ propensity to talk about treatment details and use of medical jargon (e.g., delay of tumour growth) distracts from prognostic discussions and exacerbates patients’ misconceptions [30,62,69,72]. 

### 4.2. Strengths and Limitations

The current study was the first to explore prognostic communication in the unique setting of SOs in advanced cancer care, uncovering how patients and consulting oncologists talk about prognostic information previously provided by the referring oncologist. Open and double coding of observational data, as well as recurrent extensive consensus discussions with a multidisciplinary team, supported the quality and reliability of the results. A limitation of this research concerns its limited generalisability, as only two hospitals in the Netherlands participated. Oncologists in other contexts might carry out SOs and/or prognostic communication differently. Furthermore, the participating patient group may not be representative of the entire population of advanced cancer patients. Lastly, we did not distinguish between prognostic communication by patients and their significant others, as those findings were often intertwined and we aimed to explore prognostic discussions in SOs comprehensively. Nevertheless, separate analyses of patients and significant others may yield additional insight into the dynamics of prognostic communication. 

### 4.3. Practical Implications and Recommendations

The results of this study corroborate the sensitivity of prognostic discussions for advanced cancer patients and consulting oncologists, as both regularly struggled to discuss prognosis straightforwardly. This yields missed opportunities for open prognostic discussions, which could lead to prognostic unawareness, misunderstandings and hampered medical decision-making among advanced cancer patients. Therefore, prognostic communication should be optimised. Current guidelines suggest that oncologists need to explicitly assess patients’ prognostic information preferences first while also considering the minority of patients that do not want to discuss life expectancy [17,18,61,66,73]. Consulting oncologists should listen actively for implicit cues and pose clarifying questions, empowering patients to verbalise their needs and ideas about prognosis. If it is desired, and depending on patients’ preferred level of specificity, consulting oncologists could offer explicit (e.g., numerical) prognostic information based on the available evidence. It should be made clear that statistics apply to groups of patients and are not specific to individuals. Consulting oncologists may provide the worst, typical and best-case scenarios of survival to balance realism with hope [18,66,73]. However, especially in the setting of SOs for advanced cancer, survival data are often lacking. This may require oncologists to carefully provide assessments based on their clinical experience. It is crucial to explain the nature and limitations of any estimate and check patients’ understanding of it [18,61,66,73]. When discussing prognosis-related topics, oncologists should clarify the relevance to patients’ life expectancy. Overall, an expert, emphatic and collaborative approach to communicating prognosis is recommended [18,73,74]. For future research, it is essential to complement current guidelines by examining which strategies to communicate prognosis benefit patients most.

Considering the triadic relationship characterising SOs, consulting oncologists could additionally explore what prognostic information patients have previously acquired from the referring oncologist. Although patients’ reports of the first opinion may be biased, it will enable consulting oncologists to assess what patients understood from it and whether that matches their own assessment. Consulting oncologists may agree with prognostic information gained from the referring oncologist or find (minor) inconsistencies, but should always ensure an honest, independent opinion if requested. Our findings, as well as previous literature, suggest that consulting oncologists are already committed to safeguarding patients’ and their own relationship with the referring oncologist [35,36]. Hence, we propose being transparent about significant inconsistent information. Consulting oncologists have the opportunity to complement, nuance or adjust patients’ perception of their prospects, given that patients might be more “ready” to hear prognosis when receiving a SO [37,38]. Helping patients understand discrepancies could prevent misconceptions, and facilitate patients’ awareness of prognosis and the uncertainties associated with it. Lastly, supporting patients in dealing with prognostic uncertainty seems vital. Consulting oncologists could provide them with a sense of control by explaining what to expect regarding the disease trajectory, offering a clear plan forward and emphasising the continuity of care [75,76,77].

## 5. Conclusions

This study substantiates advanced cancer patients’ need for and oncologists’ cautiousness about prognostic discussions during SO consultations. Barriers to prognostic communication seem heightened in this sensitive setting, yet the relevance of transparent conversations about prognosis might be too. In order to overcome missed opportunities, it is essential for consulting oncologists to explicitly explore patients’ information preferences and perception of their prognosis. If it is desired, consulting oncologists may provide tailored, independent information to optimise patients’ prognostic awareness and well-informed medical decision-making. Additionally, offering patients support in dealing with prognosis and the uncertainties associated with it seems valuable.
